# Supplementary material for: The innate immune sensor IFI16 recognizes foreign DNA in the nucleus by scanning along the duplex
Source: eLife. 2015 Dec 16;4:e11721. doi: 10.7554/eLife.11721 (PMC4829420; doi:10.7554/eLife.11721)
Supplement: Supplementary file 1. — (A) dsDNA-mediated oligomerization rates of FRET-labeled IFI16. (B) Oligonucleotides used in this study. DOI: http://dx.doi.org/10.7554/eLife.11721.019 [file elife-11721-supp1.docx]

**Supplementary file 1A**

dsDNA-mediated oligomerization rates of FRET-labeled IFI16. Each experiment was performed at least three times and errors were calculated by using the standard deviations.

| dsDNA size (bps) | 25 nM IFI16 (sec^-1^) | 50 nM IFI16 (sec^-1^) |
| --- | --- | --- |
| 60 | 0.0004 ± 0.0002 | 0.0009 ± 0.0002 |
| 70 | 0.0008 ± 0.0002 | 0.0021 ± 0.0008 |
| 100 | 0.0052 ± 0.0008 | 0.012 ± 0.005 |
| 150 | 0.019 ± 0.004 | 0.037 ± 0.008 |

| 200 | 0.021 ± 0.007 | 0.049 ± 0.011 |
| --- | --- | --- |
| 300 | 0.029 ± 0.008 | 0.045 ± 0.014 |
| 600 | 0.021 ± 0.008 | 0.043 ± 0.012 |

**Supplementary file 1B**

Oligonucleotides used in this study are listed below.

| oligo 1 | 5’GGGCGGCGACCTGGACAGCAAGTTGGACAATCTCGTTCTATCACTAATTCACTAATGCAGGGAGGATTTCAGATATGGCA-3’ |
| --- | --- |
| oligo 2 | 5’-biotin-A(16)GAGTACTGTACGATCTAGCATCAATCACAGG  GTCAGGTTCGTTATTGTCCA-3’ |
| oligo 3 | 5’-AGGTCGCCGCCCA(12)-biotin-3’ |
| 601-sequence | 5’-biotin-ATCGAGAATCCCGGTGCCGAGGCCGCTCAATTGGTCGT AGACAGCTCTAGCACCGCTTAAACGCACGTACGCGCTGTCCCCCGCGTTTTAACCGCCAAGGGGATTACTCCCTAGTCTCCAGGCACGTGTCAGATATATACATCCGAT |
