# Supplementary material for: The innate immune sensor IFI16 recognizes foreign DNA in the nucleus by scanning along the duplex
Source: eLife. 2015 Dec 16;4:e11721. doi: 10.7554/eLife.11721 (PMC4829420; doi:10.7554/eLife.11721)
Supplement: Source code 1. — DOI: http://dx.doi.org/10.7554/eLife.11721.020 [file elife-11721-code1.docx]

**Supplementary file 2**

Code for random walk simulation written in Python

import numpy as np

import random

import math

import matplotlib.pyplot as plt

# Diffusion coefficient d (um2/s)

d=np.double(0.026)

# Number of random walks n

n=np.double(10000)

# Simulation time t (s)

t=np.double(1000)

# Step size dt (s)

dt=t/(n-1)

tv=np.linspace(0,t,n)

# Segment length l (um)

def rnwlk(l):

x = np.zeros((2,n)) + np.random.uniform(0,l,(2,1))

s = math.sqrt(d*dt)*(np.random.randn(2,n-1))

# add vdt to steps s for introducing flow bias

# 0.01 µm distance == dimerization

x[:,1:] = x[:,1:] + np.cumsum(s,1)

try:

bmin = np.min(np.nonzero(np.mod(np.absolute(x[0]-x[1]),l) < 0.010))

tsearch = tv[bmin]

except:

tsearch=np.nan

return (x,tsearch)

searchtimes = np.zeros((10000,1))

for i in range(searchtimes.shape[0]):

(x,tsearch) = rnwlk(l)

searchtimes[i] = tsearch

searchtimesperc=np.percentile(searchtimes[np.isnan(searchtimes)==False],(2.5,50,97.5))

plt.hist(searchtimes[np.isnan(searchtimes)==False],bins=math.sqrt(n),normed=True, cumulative=True)

plt.title("2.5%: " + str(searchtimesperc[0])+" 50.0%: "+str(searchtimesperc[1])+" 97.5%: "+str(searchtimesperc[2]) )

plt.ylabel('Cumulative probability density')

plt.xlabel('Search time (s)')

plt.show()
